# Sprouty1 is a weight-loss target gene in human adipose stem/progenitor cells that is mandatory for the initiation of adipogenesis

**DOI:** 10.1038/s41419-019-1657-3

**Published:** 2019-05-28

**Authors:** Markus Mandl, Sonja A. Wagner, Florian M. Hatzmann, Maria C. Mitterberger-Vogt, Marit E. Zwierzina, Monika Mattesich, Werner Zwerschke

**Affiliations:** 1 Division of Cell Metabolism and Differentiation Research, Research Institute for Biomedical Aging Research, University of Innsbruck, Rennweg 10, A-6020 Innsbruck, Austria; 2 Department of Plastic and Reconstructive Surgery, Innsbruck Medical University, Anichstraße 35, A-6020 Innsbruck, Austria; 3 Center for Molecular Biosciences Innsbruck (CMBI), University of Innsbruck, Innrain 80-82, A-6020 Innsbruck, Austria

**Keywords:** Ageing, Stem-cell research

## Abstract

The differentiation of adipose stem/progenitor cells (ASCs) into adipocytes contributes to adipose tissue expansion in obesity. This process is regulated by numerous signalling pathways including MAPK signalling. In the present study, we show that weight loss (WL) interventions induce upregulation of Sprouty1 (*SPRY1*), a negative regulator of MAPK signalling, in human ASCs and elucidate the role of the Sprouty1/MAPK interaction for adipogenic differentiation. We found that the Sprouty1 protein levels are low in proliferating ASCs, increasing in density arrested ASCs at the onset of adipogenic differentiation and decreasing in the course of adipogenesis. Knock-down (KD) of Sprouty1 by RNA interference led to elevated MAPK activity and reduced expression of the early adipogenic transcription factor CCAAT/enhancer-binding protein β (*C/EBP β*), concomitant with an abrogation of adipogenesis. Intriguingly, co-treatment of Sprouty1 KD ASCs with differentiation medium and the pharmacological MEK inhibitor U0126 blunted ERK phosphorylation; however, failed to rescue adipogenic differentiation. Thus, the effects of the Sprouty1 KD are not reversed by inhibiting MAPK signalling although the inhibition of MAPK signalling by U0126 did not prevent adipogenic differentiation in wild type ASCs. In conclusion, we show that Sprouty1 is induced after WL in ASCs of formerly obese people acting as a negative regulator of MAPK signalling, which is necessary to properly trigger adipogenesis at early stages by a C/EBP β dependent mechanism.

## Introduction

Obesity is a major public health problem worldwide associated with several comorbidities and a reduced expectation of life^1,2^. The expansion of white adipose tissue (WAT) is a key event in obesity. It acts as endocrine and metabolic regulator buffering nutrient availability and demand by storing excess calories in form of triglycerides and delivering free fatty acids during fasting^3^. WAT is highly plastic and its enlargement is a consequence of an increased number and volume of adipocytes^4,5^. The number of adipocytes is determined by the renewal and differentiation capacity of adipose stem/progenitor cells (ASCs), which constitute a subpopulation of the adipose stromal cell pool^4,5^.

ASCs can differentiate into mature adipocytes in a process referred to as adipogenesis^5,6^. Adipogenesis is orchestrated by a complex network of transcription factors, most important are the two adipogenic factors nuclear receptor peroxisome proliferator-activated receptor *γ*2 (PPAR γ2) and CCAAT/enhancer-binding protein α (C/EBP α), which control the entire terminal differentiation process^7^. At early stages of adipogenic differentiation the expression of these transcription factors is induced by the integration of multiple anti- and pro-adipogenic signals including hormones and growth factors, such as insulin and insulin-like growth factor 1 (IGF-1)^8^. Insulin and IGF-1 can bind to appropriate tyrosine kinase receptors at the cell-surface and initiate down-stream signalling cascades, most important phosphoinositide 3 kinase (PI3K)/protein kinase B (Akt) signalling and the RAS/Mitogen-activated protein kinase (MAPK) pathway^9^. Activation of the MAPK pathway leads to extracellular signal-regulated kinase (ERK) phosphorylation and nuclear translocation^10,11^. Subsequently, transcription factors regulating cellular functions such as proliferation and differentiation are activated^10^. MAPK signalling regulates the gene expression^12^ and transcriptional activity of C/EBP β, which can act as inhibitor of cell division in the G1 phase of the cell cycle by C/EBP α-dependent^13^ and -independent mechanisms^14^ and as activator of terminal adipogenesis via induction of the expression of *C/EBP* α and *PPAR γ2*^15–17^. The MAPK cascade is negatively regulated by Sprouty (*SPRY*) proteins. Four different isoforms (Sprouty1–4) have been described in humans^11^. Sprouty1, −2 and −4 are the predominant Sprouty proteins^18^ and ubiquitously expressed in the developing embryo as well as in adult tissues^11,19^. In contrast, Sprouty3 shows a tissue restricted expression pattern (i.e., brain and testis)^11,19^ and plays a minor role in the regulation of the MAPK pathway^18^. *SPRY* expression is induced upon MAPK signalling thus forming a negative feed-back loop^20^. Sprouty1 is regarded to intercept MAPK signal transduction at early steps, whereas the exact mechanism is elusive and cell context-dependent^11^.

To treat obesity, weight-loss (WL) interventions, such as caloric restriction (CR) and bariatric surgery, are frequently applied^21,22^. These interventions postpone age-related diseases and improve health span in humans^1^ and non-human primates^23,24^. Mechanisms on how WL improves health span are a current focus of obesity and aging research^1,4,25^. To better understand the effects of WL on ASCs we compared ASCs from abdominal sWAT of normal weight (NWD), obese (OD) and formerly obese donors after weight-loss (WLDs) and showed that WL reduced the adipogenic activity in these cells^26^. Moreover, a microarray analysis was performed by our laboratory comparing global gene expression pattern in ASCs of formerly obese-, normal-weight and obese donors^27^. This work identified the small GTPase, GTP-binding RAS-like 3 (DIRAS3), as a negative regulator of PI3K/Akt signaling and adipogenesis in ASCs^27^. This suggests that the insulin/IGF-1 signalling network is an important mediator of the effects of WL interventions on ASCs.

Interestingly, in our screening for WL target genes^27^, we identified another regulator of IGF-1 signalling, Sprouty1, which was upregulated after WL in ASCs of formerly obese people. The objective of the current study was to investigate the role of Sprouty1 in adipogenic differentiation of human ASCs. By employing a gene-silencing approach this study highlights Sprouty1 to be a novel regulator of adipogenesis.

## Methods and materials

### Ethics declaration and donor characteristics

Subcutaneous white adipose tissue (sWAT) samples were obtained from patients undergoing routine elective plastic abdominal surgery at the Institute for Plastic and Reconstructive Surgery (Medical University of Innsbruck, Austria). All patients gave their informed written consent. The study protocol was approved by the Ethics Committee of the Medical University of Innsbruck (Austria) according to the Declaration of Helsinki. sWAT samples taken from the lower abdomen (infraumbilical) of *n* = 18 different donors of Caucasian origin were used. The tissue was resected from the layer between fascia of scarpa and rectus fascia. Donor characteristics are provided in Table 1.

**Table 1:** Clinical data

Donor	Sex	Age [years]	BMI [kg/m^2^]	Treatment group
1	f	28	24,22	WLD
2	f	59	27,22	WLD
3	f	62	24,35	NWD
4	m	n. a.	n. a.	n. a.
5	f	38	24,45	WLD
6	m	18	24,49	WLD
7	f	34	25,95	WLD
8	f	55	25,59	WLD
9	f	36	24,98	WLD
10	f	27	20,2	WLD
11	f	55	26,03	WLD
12	f	45	22,27	WLD
13	f	38	24,61	NWD
14	f	23	19,83	NWD
15	f	29	23,71	NWD
16	f	44	31	OD
17	f	48	33,06	OD
18	f	48	31,99	OD

Human sWAT samples were taken from the lower abdomen from healthy donors undergoing routine abdominoplasty at the Institute for Plastic and Reconstructive Surgery at the Medical University of Innsbruck, Austria

*BMI* body mass index, *NWD* normal weight donor, *OD* obese donor, *WLD* weight loss donor, *f* female, *m* male, n.a. not available

### Isolation of human Adipogenic stromal/progenitor cells (ASCs) and cell culture

Human ASCs were isolated from abdominal sWAT obtained from patients undergoing routine elective plastic abdominal surgery at the Institute for Plastic and Reconstructive Surgery at the Medical University of Innsbruck (Austria). The adipose tissue was processed according to a well-established protocol^28^. Briefly, the tissue samples were washed with PBS and dissected under sterile conditions. Connective tissue and blood vessels were removed. Collagenase digestion was performed (PBS containing 200 U/ml collagenase (CLS Type I, Worthington Biochemical Corp., Lakewood, NJ) and 2% w/v BSA) under stirring for 60 min at 37 °C (1 mg adipose tissue/3 ml digestion solution). Subsequently, samples were centrifuged for 10 min at 200RCF. The cell pellet was re-suspended in erythrocyte lysis buffer (0.155 M NH_4_CI, 5.7 mM K_2_HPO_4_, 0.1 mM EDTA, pH 7.3), incubated for 10 min at room temperature followed by filtration through a cell-strainer (pore size 100 µm). Cells were centrifuged as above and the resulting stromal vascular fraction (SVF) was re-suspended in ASC medium (DMEM/F-12 medium with HEPES and L-Glutamine (purchased from Sigma Aldrich, Vienna, Austria or Gibco, Vienna, Asutria) containing 33 μM Biotin, 17 μM Pantothenate, 20 μg/ml Ciprofloxacin) supplemented with 10% FCS (Gibco, Vienna, Austria). SVF cells were filtered through a cell-strainer with 35 µm pore size and seeded into six-well plates at a density of 70.000 cells/cm^2^. Cells were allowed to attach overnight followed by culture in serum-free ASC medium for six days (passage 1) under canonical conditions (37 °C, 5% CO_2_, humidified atmosphere). ASCs were harvested by trypsinization and stored in liquid nitrogen or maintained in PM4 medium (ASC medium supplemented with 2.5% FCS, 10 ng/ml EGF, 1 ng/ml bFGF, 500 ng/ml Insulin). ASCs were sub-cultured at 70% confluence in a ratio of 1:2 using ASC medium containing 10% FCS. On the next day, the supernatant was replaced by PM4 medium.

### Cloning procedures

To knock down Sprouty1, a set of five pLKO.1 plasmids encoding different shRNAs targeting the human *SPRY1* gene were purchased from a commercial supplier (Dharmacon™, TRCN00000 5693-3 to -7; in this study: TRCN00000 5693-5 is referred to as shRNA#1, -6 is referred to as shRNA#2). For comparison, a non-targeting control was employed^27^. For ectopic overexpression of Sprouty1, an appropriate pENTR223 plasmid containing the human *SPRY1* cDNA was obtained from the DNASU Plasmid Repository (HsCD00288035) and cloned into the pLenti6/V5-DEST vector by recombination using the Gateway® System (Invitrogen) as described in the manufacturer´s guidelines. For control, the empty vector was used^29^. All plasmids were amplified in *E. coli Stbl3* bacteria. Endotoxin-free plasmid preparations for transfection were gained using the EndoFree® Plasmid MaxiKit (Qiagen) according to the manufacturer´s protocol.

### Generation of lentiviral particles and infection of ASCs

Lentiviral particles for gene transduction were produced in HEK293FT cells and titrated as previously described^27^. The particles were stored at −80 °C. ASCs were seeded in 175 cm^2^ flasks in growth medium containing 10% FCS at a density of ~3 × 10^3^-4.5 × 10^3^ cells/cm^2^ and allowed to adhere overnight. On the next day (morning), the supernatant was discarded and replaced by PM4 medium. Followed an incubation period of approximately 6 h, ASCs were infected using a multiplicity of infection (MOI) of 4. For this purpose, the appropriate amount of viral particles for one 175 cm^2^ flask was diluted in 15 ml PM4 medium containing 6 µg/ml Polybrene. The culture medium was removed and the virus applied onto the ASCs overnight. Subsequently, the supernatant was discarded and replaced by 25 ml PM4 medium. Cells were allowed to recover for up to 3 days post-infection, followed by antibiotic selection (pLKO.1: Puromycin: 2 µg/ml in PM4 medium; pLenti6: Blasticidin: 10 µg/ml in PM4 medium) for at least 3 days. Transduced ASCs were expanded in PM4 medium.

### Differentiation of ASCs into adipocytes

Adipogenic differentiation of ASCs was carried out as described^30^.

### U0126 Treatment

The MEK inhibitor U0126 was purchased from Merck Calbiochem (#662005), dissolved in DMSO (stock concentration 5 mM) and stored at −20 °C. For ASC treatment, U0126 was added into the differentiation medium at a final concentration of 10 µM. DMSO was used as vehicle control.

### Western blot analysis

To obtain protein samples for Western blotting, cells were washed with PBS and lysed with a SDS sample buffer, followed by the determination of protein concentration as previously described^31^. A total amount of 10 µg protein per sample was dissolved on an acrylamide gel (8–12.5 %) and wet-blotted onto a PVDF membrane (Perkin Elmer). Membranes were probed with primary antibodies (Supplementary Table 1) overnight at 4 °C. To ensure equal loading of samples, PVDF membranes were incubated with an β-Actin antibody (1:100.000; Sigma Aldrich, AC-15, #A5441) for 1 h at room temperature. Appropriate secondary HRP-conjugated antibodies (Anti-Mouse IgG, #W402B, Promega; Polyclonal Swine Anti-Rabbit IgG, #P0399, DAKO) were diluted 1:5000 and applied for 1 h at room temperature. Signal development was achieved with the Western Lightning® Plus ECL reagent (Perkin Elmer). Finally, PVDF membranes were exposed to a medical X-ray film (Super RX-N, FUJI). Quantification of signals was performed using ImageJ software (version 1.47, National Institutes of Health, USA).

### Gene expression analysis

Gene expression was analysed as described previously^31^. The RNeasy Plus Micro Kit (Qiagen, #74034) was used to isolate total RNA in accordance with the manufacturer´s protocol. cDNA synthesis was performed using the First Strand cDNA Synthesis Kit (Thermo Scientific, #K1622) as described in the supplier´s guidelines. Primer sequences for quantitative real-time Polymerase chain reaction (qPCR) are given in Supplementary Table 2. Gene expression was measured with a LightCycler® 480 (Roche) instrument using SYBR green chemistry (LightCycler® 480 SYBR Green I Master, Roche Life Science or AceQ qPCR SYBR Green, Vazyme Biotech). Comparative relative quantification (ΔΔC_T_ method) was calculated with β-Actin (*ACTB*) expression as endogenous control.

### Microarrays

Microarrays were done in Ejaz et al. 2016^27^.

### Quantification of intracellular lipids

Intracellular lipids were stained with Oil Red O (ORO) as described in ref. ^30^. For quantification, ORO was re-dissolved with 1 ml Isopropanol for 30 min and absorbance was measured at 570 nm.

### Statistics

Statistical analysis was performed with GraphPad Prism 5 software (GraphPad Software Inc., La Jolla, CA, USA). Each experiment was conducted with a minimum of *n* = 3 biological replicates (i.e., donors). All measurements were done in triplicates. Values are given as mean ± SEM. Statistical comparison was achieved using the unpaired two-tailed *t*-test or ANOVA depending on the type of the data set and as indicated in the corresponding figure legend. *p* values ≤ 0.05 were considered to be significant.

## Results

### *SPRY1* expression is up-regulated upon WL in human ASCs

**Fig. 1:**
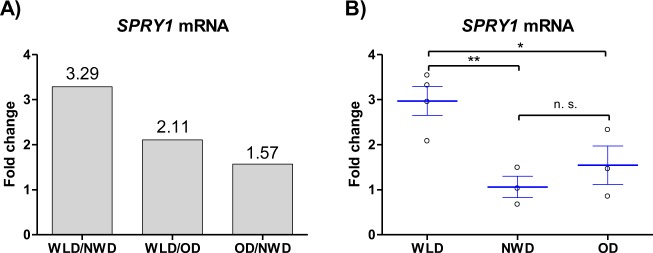
*SPRY1* expression is upregulated upon weight loss (WL) in human ASCs. **a** Microarray profiling of freshly isolated ASCs was carried out as already described in Ejaz et al. 2016^27^. Tissue samples were obtained from aged-matched Normal weight donors (NWD; *n* = 3), Obese donors (OD; *n* = 3) and donors subjected to a weight loss intervention (Weight loss donors: WLD; *n* = 4). **b** Sprouty1 expression relative to β-actin was analysed by RT-qPCR in early passage ASCs derived from WLDs (*n* = 4), NWDs (*n* = 3) and ODs (*n* = 3) to verify the Microarray

Sprouty1 was one WL target gene which emerged from a global gene expression analysis previously performed in our laboratory on human ASCs freshly isolated from subcutaneous White-adipose tissue (WAT). To do this, cells from age and sex matched WL donors (WLDs), NWDs and ODs were subjected to whole genome microarray gene expression analysis (Affymetrix Chip U133 + 2.0)^27^. Results revealed a 3.29-fold higher expression of Sprouty1 in ASCs of WLDs relative to NWDs and a 2.11-fold higher expression of Sprouty1 in ASCs from WLDs relative to ODs (Fig. 1a). Quantitative real time PCR confirmed a significant up-regulation of Sprouty1 expression in early passage of ASCs of the WLDs relative to NWDs and ODs (Fig. 1b). We conclude, Sprouty1 is up-regulated upon WL in human ASCs.

### Sprouty1 expression during Adipogenesis

**Fig. 2:**
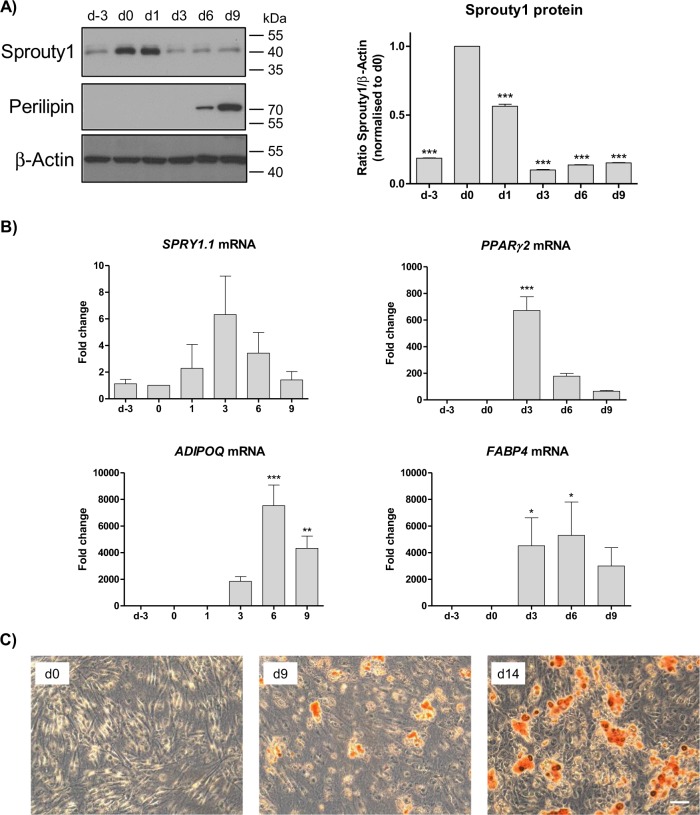
Sprouty1 *(SPRY1)* expression during adipogenesis. **a** Western blot analysis of sprouty1 and perilipin levels in ASCs. Left panel: Adipogenesis was induced on d0. β-actin served as input control. Molecular masses are given in kDa. Right panel: Densitometric quantification of the Western blot. Values are expressed as mean ± SEM, *n* = 3. Statistical comparison to d0 was achieved using one-way ANOVA and Dunnett´s Multiple Comparison test. **b** mRNA expression analysis of *SPRY1.1*, *PPAR γ2*, adiponectin (*ADIPOQ*) and *FABP4* using RT-qPCR in differentiating ASCs. Values are expressed as mean ± SEM, *n* = 3. Statistical comparison to d0 was achieved using ANOVA. **c** Oil Red O staining of ASCs is shown prior the induction of differentiation (d0), on d9 and d14 of adipogenesis. Magnification ×10. Scale bar 100 µm. In **a**–**c** representative results from one out of four donors are shown

To elucidate the function of Sprouty1 in human ASCs we first monitored its expression in the course of adipogenic differentiation. Western blot analysis revealed a strong upregulation of Sprouty1 protein in growth arrested confluent cells (d0) relative to proliferating cells (d-3). The Sprouty1 protein level declined after the onset of adipogenesis (Fig. 2a). Sprouty1 (*SPRY1.1*) mRNA expression was not significantly altered during adipogenesis (Fig. 2b). Adipogenic differentiation was confirmed by the expression of *PPARγ2*, Adiponectin and *FABP4* mRNA, increasing levels of Perilipin protein and the formation of lipid droplets (Fig. 2b, c). We conclude, the Sprouty1 protein levels are strongly increasing in ASCs after transition from proliferation to growth arrest, stay high at early adipogenesis and decreasing in the course of differentiation.

### Knock-down of Sprouty1 inhibits Adipogenesis

**Fig. 3:**
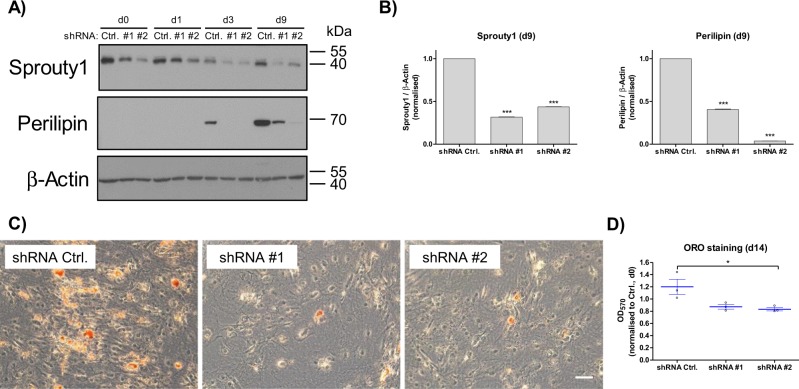
Knock-down of Sprouty1 in ASCs inhibits adipogenic differentiation. **a** Western blot analysis of Sprouty1 and perilipin protein levels in Sprouty1 KD ASCs. A non-targeting control shRNA (Ctrl.) and two Sprouty1-targeting shRNAs (shRNA #1 and #2) were employed. Adipogenesis was induced on d0. β-actin served as input control. Molecular masses are given in kDa. **b** Densitometry of the Western blot shown in **a**) (corresponding time-point d9). Values are expressed as mean ± SEM, *n* = 3. Statistical comparison to the appropriate control was achieved using the two-tailed paired *t*-test. **c** Oil Red O staining of differentiated ASCs on d14. Representative example of *n* = 3 independent experiments (i.e., donors). Magnification ×10. Scale bar 100 µm. **d** Photometric quantification of Oil Red O staining (d14) from *n* = 3 different donors. Values are expressed as mean ± SEM. Statistical comparison was done using the two-tailed unpaired *t*-test

To understand the high abundance of Sprouty1 protein in confluent ASCs at the onset of adipogenic differentiation, we investigated the effects of Sprouty1 depletion in this context. For this purpose, a gene-silencing approach using shRNAs was employed to KD Sprouty1 in ASCs (Fig. 3a, b). This lead to an attenuation of adipogenic differentiation, as shown by strongly reduced protein levels of Perilipin and impaired lipid accumulation in Sprouty1-depleted ASCs in the course of differentiation (Fig. 3c, d). *SPRY1* overexpression had no statistically significant effect on adipogenesis (Supplementary Fig. S1). These results suggest that the presence of Sprouty1 is crucial for the onset of adipogenesis.

### Sprouty1 depletion augments ERK signalling and prevents *C/EBP* β expression

**Fig. 4:**
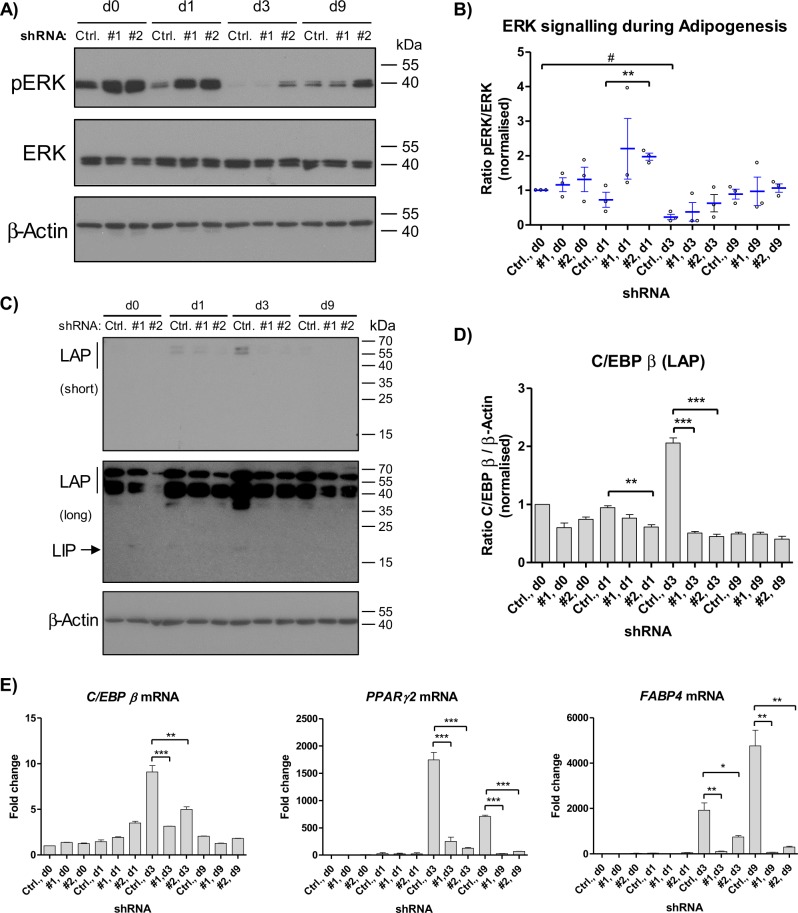
Effects of Sprouty1 KD on adipogenesis. **a** Western blot analysis of ERK activation in differentiating Sprouty1 KD ASCs. β-actin served as input control. Molecular masses are given in kDa. Representative result of *n* = 3 independent experiments (i.e., donors). **b** Ratio of pERK/ERK during adipogenesis in Sprouty1 KD ASCs derived from *n* = 3 different donors. Values are expressed as mean ± SEM. Statistical comparison was done using the two-tailed unpaired (indicated by *) or the paired (indicated by #) *t*-test. **c** Western blot analysis of C/EBP β (LAP and LIP isoforms) in differentiating Sprouty1 KD ASCs. β-actin served as input control. Molecular masses are given in kDa. Representative result of *n* = 3 independent experiments (i.e., donors). short: short exposure (90 sec); long: long exposure (1 h); **d** Densitometric quantification of the LAP isoform corresponding to the result shown in **c**). Values are expressed as mean ± SEM, *n* = 3. Statistical comparison was done using the two-tailed unpaired *t*-test. **e** mRNA expression analysis using RT-qPCR of key factors during adipogenic differentiation as indicated. Representative result of *n* = 3 independent experiments (i.e., donors). Values are expressed as mean ± SEM. Statistical comparison was done employing the two-tailed unpaired *t*-test

Sprouty1 is a known negative regulator of MAPK signalling^20^. Therefore, it was tested whether silencing of Sprouty1 affects MAPK activation. As expected, Western blot analysis revealed a higher phosphorylation and hence activation of ERK in the Sprouty1 KD ASCs, predominantly observed at day 1 after induction of differentiation (Fig. 4a). This is reflected by the higher pERK/ERK ratio in the Sprouty1 KDs compared to control cells (Fig. 4b). Given the tight regulation of C/EBP β by the Ras/MAPK pathway^12,15^, the expression of this early adipogenic transcription factor was analysed. As shown in Fig. 4c, d, the induction of C/EBP β (Full-LAP, LAP and LIP isoforms) during adipogenesis was inhibited in Sprouty1-depleted ASCs. Intriguingly, RT-qPCR analysis showed a decreased mRNA expression of *C/EBP* β and its target gene *PPARγ2* in Sprouty1 depleted ASCs during adipogenesis (Fig. 4e). *FABP4* induction was also impaired in Sprouty1 KD ASCs compared to control. These results indicate that silencing of Sprouty1 prevents adipogenesis by inhibiting *C/EBP* β expression.

### Inhibition of MAPK signalling by U0126 doesn’t prevent terminal adipogenic differentiation

**Fig. 5:**
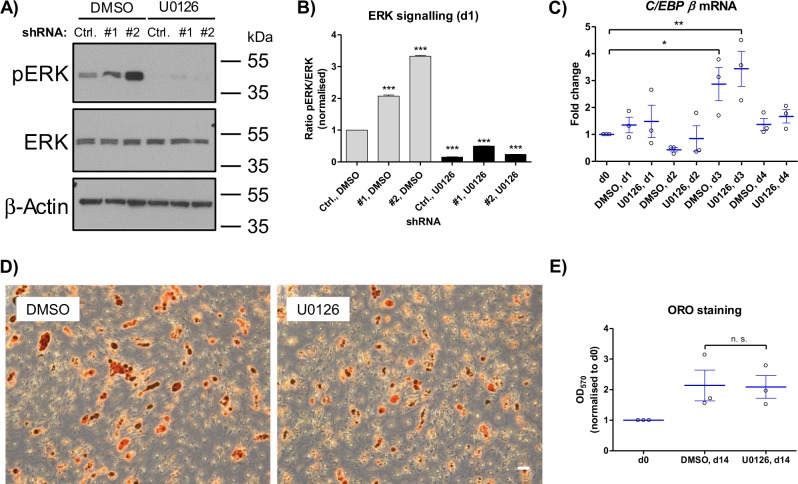
Effects of the MEK inhibitor U0126 on adipogenesis in ASCs. **a** Western blot analysis of ERK activation in differentiating Sprouty1 KD ASCs co-treated with DMSO (vehicle control) or U0126 (10 µM) for 1 day. β-actin served as input control. Molecular masses are given in kDa. Representative result of *n* = 3 independent experiments (i.e., donors). **b** Densitometry corresponding to **a**). Values are expressed as mean ± SEM, *n* = 3. Statistical comparison was done using one-way ANOVA and Dunnett´s Multiple Comparison test. **c** RT-qPCR analysis of wildtype ASCs treated with DMSO (vehicle-control) or U0126 (10 µM) during adipogenesis. Values are presented as mean ± SEM of *n* = 3 different donors. Statistical comparison was done using one-way ANOVA and Dunnett´s Multiple Comparison test. **d** Oil Red O staining of DMSO or U0126 (10 µM) treated ASCs on d14 of adipogenic differentiation. Representative result of n = 3 different donors. Magnification ×5. Scale bar 100 µm. **e** Photometric quantification of Oil Red O staining on d14 of adipogenesis. Corresponding to the results shown in D). Values are presented as mean ± SEM of *n* = 3 different donors. Statistical comparison was performed using the two-tailed unpaired *t*-test. n. s.: not significant

Since the Sprouty1 KD strongly increased MAPK signaling and inhibited adipogenesis (Fig. 4), we asked whether these effects might be reversed by inhibiting the MAPK signalling pathway using the pharmacological MEK inhibitor U0126^32^. Co-treatment of Sprouty1 KD ASCs with differentiation medium and 10 µM U0126 blunted ERK phosphorylation (Fig. 5a, b) but failed to rescue adipogenic differentiation (data not shown). Hence, we tested whether terminal adipogenesis per se is susceptible to MAPK inhibition in human ASCs. To do this, adipogenesis was induced in non-transduced ASCs in the presence and absence of 10 µM U0126. As shown in Fig. 5c, *C/EBP* β expression was normally induced in both untreated and U0126-treated ASCs. Moreover, staining of intracellular lipid accumulation by Oil Red O on d14 after induction of adipogenesis revealed no significant effect of U0126 on terminal adipogenic differentiation. Thus, pharmacological inhibition of MAPK signalling by U0126 does not prevent terminal adipogenic differentiation of primary human ASCs. This is in agreement with our finding that MAPK signalling per se is reduced during adipogenesis (see above, Fig. 4).

## Discussion

Adipogenesis is a highly orchestrated process depending on several signalling pathways^7,33^. Among these pathways, the MAPK cascade plays an important role. The impact of MAPK on adipogenic differentiation has been intensively studied in murine 3T3-L1 pre-adipocytes, a model system in which adipogenic differentiation runs in two stages, clonal expansion (proliferation) and terminal adipogenic differentiation^17^. Studies performed in this cell type demonstrated that ERK signalling is required for clonal expansion and must afterwards be terminated to enable terminal adipogenic differentiation^34,35^. In fact, in 3T3-L1 cells ERK activity promotes the expression of adipogenic factors, such as PPARγ, whereas ERK-mediated phosphorylation of PPARγ inhibits its transcriptional activity and hence adipogenesis^36,37^. In contrast, the importance of ERK activity for terminal adipogenesis in primary human ASCs, which differentiate directly into adipocytes without clonal expansion, is not precisely understood^8,38^.

Sprouty1, a negative regulator of MAPK signalling^39^, has been implicated in the maintenance of stem cells^40,41^. We identified Sprouty1 as a gene induced by WL interventions in human ASCs and elucidated the role of Sprouty1 for MAPK signalling and adipogenic differentiation in these cells as an ex vivo model^31,42–44^. Sprouty1 was highly abundant at the onset of adipogenesis and decreased later on. Depletion of Sprouty1 hyper-activated MAPK signalling and inhibited the expression of *C/EBP* β, a key transcription factor acting upstream of the adipogenic cascade^17^. In accordance with our work, there is precedence that hyper-activation of MAPK downregulates *C/EBP* β gene expression^12^. It was shown that overexpression of the constitutive active Ras^V12^ mutant downregulated *C/EBP* β mRNA expression in the immortalized NIH 3T3 cell line^12^.

Due to the observation that Sprouty1 KD increased ERK phosphorylation and inhibited *C/EBP* β expression, we tested whether both effects might be reversed by pharmacological inhibition of the MAPK pathway. Treatment of Sprouty1 KD ASCs with the MEK inhibitor U0126 blunted ERK phosphorylation but had no significant effect on *C/EBP* β gene expression in these cells. In addition, U0126 treatment of differentiating wild type ASCs had no effect on *C/EBP* β expression and adipogenesis. The U0126 response in our human ASCs is in agreement with a previous study performed in human bone marrow stromal cells (BMSCs)^45^. U0126 treatment of BMSCs subjected to adipogenic differentiation did not influence *C/EBP* β expression but slightly impaired adipogenesis as judged by Oil Red O staining in this model^45^. Moreover, the authors demonstrated that controlling the magnitude and temporal activation of ERK is crucial for the adipogenic-osteogenic cell fate decision^45^. Thus, we and others show that inhibition of MAPK signalling doesn’t prevent adipogenic differentiation in human ASCs and BMSCs. In contrast to human ASCs, adipogenesis was blocked by U0126 in murine 3T3-L1 pre-adipocytes due to the inhibition of clonal expansion^35^, a major difference between both models^38^.

We identified Sprouty1 as a gene induced by WL interventions, including CR, in human ASCs. CR, also referred to as dietary restriction, is defined as lessening food intake (typically by about 30% in rodents) without malnutrition and has been demonstrated to extend the healthy life span in model organisms ranging from invertebrates to monkeys^23,24,46,47^. The longevity effect of CR is not completely understood. However, research of the last decades has shown that reduced insulin/IGF-1 signal transduction mediates beneficial effects of CR. While the importance of reduced signalling through the PI3K/Akt/mTOR down-stream signalling cascade of the Insulin/IGF-1 receptor is well understood, more recent studies suggest that beneficial CR effects are also mediated by the reduction of Ras/MAPK signalling^48^. Our data support the concept that reduced Ras/MAPK signalling, as WL response mediated by the induction of Sprouty1, confers a protective effect by properly regulating adipogenic differentiation in fasting individuals. An excess of calorie intake after WL might override this barrier and promote adipogenesis. Studies in conditional Sprouty1 knockout mice support this model^49^. They showed that adipose-specific deletion of Sprouty1 leads to high body fat accumulation and low bone mass^49^. This phenotype was reversed by a Sprouty1 gain-of-function approach. Moreover, the authors concluded that Sprouty1 might act on C/EBP β regulation^49^. In keeping with this notion, our own data indicate that Sprouty1 modulates adipogenesis upstream of C/EBP β. In another study by Urs et al.^50^, it was demonstrated that Sprouty1 expression confers a protective effect in reducing hyperplasia and hypertrophy of adipose tissue in mice subjected to a high-fat diet^50^. This finding supports our finding that *SPRY1* expression is upregulated after weight loss in formerly obese donors.

In conclusion, our study shows that Sprouty1 abundance is crucial for the onset of adipogenesis, which suggests that MAPK signalling needs to be down-regulated at the initiation phase. Our data indicate that Sprouty1 acts upstream of C/EBP β in differentiating human ASCs and needs to be tightly balanced to prevent MAPK over-activation. This study highlights Sprouty1 to be a key molecule in adipogenesis, which might provide opportunities for future therapeutic interventions to treat obesity.

## Supplementary information

Supplementary Figure S1

Supplementary Table 1

Supplementary Table 2

Supplementary figure legends
