# Supplementary figures and images for: Sprouty1 is a weight-loss target gene in human adipose stem/progenitor cells that is mandatory for the initiation of adipogenesis
Source: Cell Death Dis. 2019 May 28;10(6):411. doi: 10.1038/s41419-019-1657-3 (PMC6538615; doi:10.1038/s41419-019-1657-3)

A)

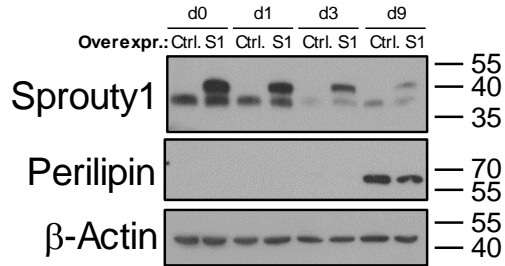

B)

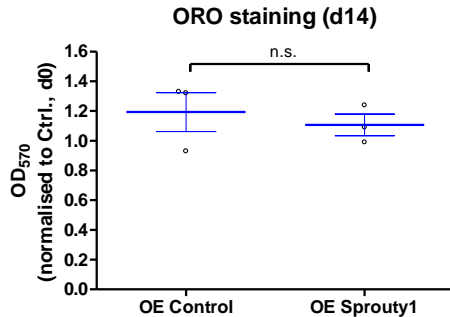

Supplement: Supplementary file 1 — Supplementary Figure S1 [file 41419_2019_1657_MOESM1_ESM.pdf]
