# Supplementary material for: Sprouty1 is a weight-loss target gene in human adipose stem/progenitor cells that is mandatory for the initiation of adipogenesis
Source: Cell Death Dis. 2019 May 28;10(6):411. doi: 10.1038/s41419-019-1657-3 (PMC6538615; doi:10.1038/s41419-019-1657-3)
Supplement: Supplementary file 2 — Supplementary Table 1 [file 41419_2019_1657_MOESM2_ESM.docx]

**Supplementary Table 1:** Antibodies for Western blotting.

| Antibody | Catalogue # | Supplier | Dilution |
| --- | --- | --- | --- |
| C/EBP β | sc-150 | Santa Cruz Biotechnology | 1:1000 |
| ERK1/2 | 9102 | Cell Signaling Technologies | 1:1000 |
| Perilipin | 9349 | Cell Signaling Technologies | 1:1000 |
| Phospho-ERK1/2 | 9101 | Cell Signaling Technologies | 1:1000 |
| Sprouty1 | 13013 | Cell Signaling Technologies | 1:1000 |
