# Supplementary material for: Sprouty1 is a weight-loss target gene in human adipose stem/progenitor cells that is mandatory for the initiation of adipogenesis
Source: Cell Death Dis. 2019 May 28;10(6):411. doi: 10.1038/s41419-019-1657-3 (PMC6538615; doi:10.1038/s41419-019-1657-3)
Supplement: Supplementary file 3 — Supplementary Table 2 [file 41419_2019_1657_MOESM3_ESM.docx]

**Supplementary Table 2:** Primer sequences.

| Primer | Sequence (5´ - 3´) | Reference |
| --- | --- | --- |
| Actin_beta_hum_for | AGAAAATCTGGCACC ACACC | [^1^](#_ENREF_1) |
| Actin_beta_hum_rev | AGAGGCGTACAGGGATAGCA | [^1^](#_ENREF_1) |
| AdipoQ_hum_for | CCTGGTGAGAAGGGTGAGAA | [^2^](#_ENREF_2) |
| AdipoQ_hum_rev | GTAAAGCGAATGGGCATGTT | [^2^](#_ENREF_2) |
| Ap2-hum-II_for | TACTGGGCCAGGAATTTGAC | [^3^](#_ENREF_3) |
| Ap2-hum-II_rev | GTGGAAGTGACGCCTTTCAT | [^3^](#_ENREF_3) |
| hCEBPb_for | AAGCACAGCGACGAGTACAA | [^3^](#_ENREF_3) |
| hCEBPb_rev | AGCTGCTCCACCTTCTTCTG | [^3^](#_ENREF_3) |
| hSpry1.1_for | GGGATTGTCCGAAAAGGATT | [^4^](#_ENREF_4) |
| hSpry1.1_rev | TTGATTTTGGGGATCCATGT | [^4^](#_ENREF_4) |
| hSPRY1_forw | ACGAGCACAGACACACAAGC | Used to confirm microarray screen |
| hSPRY1_rev | GGAACCCTTCAAGTCATCCA |  |
| PPARg2_hum_for | ATGGGTGAAACTCTGGGAGA | [^5^](#_ENREF_5) |
| PPARg2_hum_rev | TGGAATGTCTTCGTAATGTGGA | [^5^](#_ENREF_5) |

**Supplementary References**

1 Ejaz, A. *et al.* Weight Loss Upregulates the Small GTPase DIRAS3 in Human White Adipose Progenitor Cells, Which Negatively Regulates Adipogenesis and Activates Autophagy via Akt-mTOR Inhibition. *EBioMedicine* **6**, 149-161, doi:10.1016/j.ebiom.2016.03.030 (2016).

2 Mitterberger, M. C., Lechner, S., Mattesich, M. & Zwerschke, W. Adipogenic differentiation is impaired in replicative senescent human subcutaneous adipose-derived stromal/progenitor cells. *The journals of gerontology. Series A, Biological sciences and medical sciences* **69**, 13-24, doi:10.1093/gerona/glt043 (2014).

3 Lechner, S., Mitterberger, M. C., Mattesich, M. & Zwerschke, W. Role of C/EBPbeta-LAP and C/EBPbeta-LIP in early adipogenic differentiation of human white adipose-derived progenitors and at later stages in immature adipocytes. *Differentiation; research in biological diversity* **85**, 20-31, doi:10.1016/j.diff.2012.11.001 (2013).

4 Felfly, H. & Klein, O. D. Sprouty genes regulate proliferation and survival of human embryonic stem cells. *Scientific reports* **3**, 2277, doi:10.1038/srep02277 (2013).

5 Ejaz, A., Mattesich, M. & Zwerschke, W. Silencing of the small GTPase DIRAS3 induces cellular senescence in human white adipose stromal/progenitor cells. *Aging* **9**, 860-879, doi:10.18632/aging.101197 (2017).
