# Supplementary material for: Sprouty1 is a weight-loss target gene in human adipose stem/progenitor cells that is mandatory for the initiation of adipogenesis
Source: Cell Death Dis. 2019 May 28;10(6):411. doi: 10.1038/s41419-019-1657-3 (PMC6538615; doi:10.1038/s41419-019-1657-3)
Supplement: Supplementary file 4 — Supplementary figure legends [file 41419_2019_1657_MOESM4_ESM.docx]

**Supplementary Figure Legend**

**Supplementary Figure S1:** Overexpression (OE) of Sprouty1 (*SPRY1*) in ASCs subjected to adipogenic differentiation. **A)** Western blot analysis of transduced ASCs. A control- (Ctrl.) and a *SPRY1*-overexpression (S1) vector was employed. Adipogenesis was induced on d0. Molecular masses are given in kDa. Representative results of n3 independent experiments with ASCs from different donors are shown. **B)** Photometric quantification of Oil Red O staining (d14) from n=3 different donors. Values are expressed as mean +/- SEM. Statistical comparison was done using the two-tailed unpaired t-test. n. s.: not significant.
